# Building a Hand-Curated ceRNET for Endometrial Cancer, Striving for Clinical as Well as Medicolegal Soundness: A Systematic Review

**DOI:** 10.3390/ncrna11030034

**Published:** 2025-04-30

**Authors:** Roberto Piergentili, Stefano Sechi, Lina De Paola, Simona Zaami, Enrico Marinelli

**Affiliations:** 1 Institute of Molecular Biology and Pathology, Italian National Research Council (CNR-IBPM), 00185 Rome, Italy; stefano.sechi@cnr.it; 2 Department of Anatomical, Histological, Forensic and Orthopedic Sciences, Sapienza University of Rome, 00161 Rome, Italy; lina.depaola@uniroma1.it (L.D.P.); simona.zaami@uniroma1.it (S.Z.); 3 Department of Medico-Surgical Sciences and Biotechnologies, Sapienza University of Rome, 04100 Latina, Italy; enrico.marinelli@uniroma1.it

**Keywords:** gene regulation network, cancer, artificial intelligence, innovative cancer therapy, ethics, medicolegal viability

## Abstract

**Background/Objectives:** Competing endogenous RNAs (ceRNA) are molecules that compete for the binding to a microRNA (miR). Usually, there are two ceRNA, one of which is a protein-coding RNA (mRNA), with the other being a long non-coding RNA (lncRNA). The miR role is to inhibit mRNA expression, either promoting its degradation or impairing its translation. The lncRNA can “sponge” the miR, thus impeding its inhibitory action on the mRNA. In their easier configuration, these three molecules constitute a regulatory axis for protein expression. However, each RNA can interact with multiple targets, creating branched and intersected axes that, all together, constitute what is known as a competing endogenous RNA network (ceRNET). **Methods:** In this systematic review, we collected all available data from PubMed about experimentally verified (by luciferase assay) regulatory axes in endometrial cancer (EC), excluding works not using this test; **Results:** This search allowed the selection of 172 bibliographic sources, and manually building a series of ceRNETs of variable complexity showed the known axes and the deduced intersections. The main limitation of this search is the highly stringent selection criteria, possibly leading to an underestimation of the complexity of the networks identified. However, this work allows us not only to hypothesize possible gap fillings but also to set the basis to instruct artificial intelligence, using adequate prompts, to expand the EC ceRNET by comparing it with ceRNETs of other cancers. Moreover, these networks can be used to inform and guide research toward specific, though still unidentified, axes in EC, to complete parts of the network that are only partially described, or even to integrate low complexity subnetworks into larger more complex ones. Filling the gaps among the existing EC ceRNET will allow physicians to hypothesize new therapeutic strategies that may either potentiate or substitute existing ones. **Conclusions:** These ceRNETs allow us to easily visualize long-distance interactions, thus helping to select the best treatment, depending on the molecular profile of each patient, for personalized medicine. This would yield higher efficiency rates and lower toxicity levels, both of which are extremely relevant factors not only for patients’ wellbeing, but also for the legal, regulatory, and ethical aspects of miR-based innovative treatments and personalized medicine as a whole. This systematic review has been registered in PROSPERO (ID: PROSPERO 2025 CRD420251035222).

## 1. Introduction

Endometrial cancer (EC) is the most frequent gynecological malignancy in developed nations, with the highest rates in North America, Europe, Micronesia/Polynesia, and Australia/New Zealand; it is the sixth most diagnosed cancer in women, with 417,000 new cases and 97,000 deaths in 2020 [1]. Risk factors for EC include metabolic alterations (obesity, metabolic syndrome, insulin resistance) [2,3], hormonal imbalance [4], age at menopause [5], reproductive factors [6], and inherited conditions, such as Lynch Syndrome [7]. Other genetic factors have been identified and accounted for, thus allowing for the classification of EC into four classes with distinct clinical, pathologic, and molecular features [8], namely POLE (polymerase epsilon)/ultramutated (7% of cases), microsatellite instability (MSI)/hypermutated (28%), copy-number low/endometrioid (39%), and copy-number high/serous-like (26%). In addition to the mutation of genes specific for the above-mentioned conditions (*POLE*, *MLH1*, *TP53*), many other mutated genes had been identified in EC patients, including *PTEN*, *PIK3CA*, *PIK3R1*, *CTNNB1*, *ARID1A*, *PPP2R1A,* and *FBXW7*, all present at a high frequency in EC patients [9]. Additional genetic causes of EC etiopathogenesis include single-nucleotide polymorphisms in specific genes [10], altered telomere length [11], and epigenetic factors, such as DNA hypermethylation of target gene promoters (e.g., *MLH1*) [12,13].

Within living organisms, the complexity of genetics is not solely determined by the quantity of DNA in a genome. There are organisms deemed less “complex” despite having a higher amount of DNA in their cells, a phenomenon known as the C-value paradox. This also extends to the G-value paradox [14,15], which refers to the number of genes in their genomes. While both values typically correlate with organismal complexity [15], this correlation is notably disrupted in evolutionarily related species that significantly vary in either or both parameters. Complexity, rather than being quantified by DNA content, is better understood as “the extent of interactions, whether orderly or disorderly, among the components of a system”. Within living systems, complexity can be defined as “the total number of gene-gene/molecular interactions accumulated throughout an organism’s lifespan in its natural habitat” [16]. In recent decades, a growing body of the scientific literature has demonstrated that only a small fraction of the genome contains protein-coding genes, with the majority of the transcriptome being non-coding in nature. These non-coding RNAs (ncRNAs) encompass a larger proportion (both in mass and sheer number of molecules) due to the contributions of tRNA and rRNA [17]. However, a notable albeit smaller fraction of ncRNAs participates in regulating gene expression. The intricate web of interactions among molecules constitutes a significant portion of an organism’s complexity, with the control of gene expression playing a pivotal role in both physiological well-being and pathological conditions.

Overall, ncRNAs are divided into long and short—lncRNA and sncRNA, respectively—based on their length, with the former exceeding 200 nucleotides (nt) and the latter being below this threshold and usually in the 20–30 nt range. In the context of post-transcriptional gene expression control, microRNAs (abbreviated as miR or miRNA) are the most broadly researched kind of sncRNA. By annealing to their targets, such molecules can inhibit mRNA expression by either promoting its degradation or by impairing its translation [18]. The high number of miR inside human cells (>2500) and their imprecise annealing (partial mismatch is permitted, within certain limits) allows them to target multiple mRNAs at the same time, making them capable of regulating over 60% of human genes [19]. Among their many effects, miR directly regulate several genes involved in cell cycle control and cell survival, making them key players in carcinogenesis [20]. In turn, these molecules may be inhibited through their binding to lncRNA, which act as sponges to lower miR intracellular availability; in this case, lncRNA behave as miR inhibitors and, consequently, they are implicated in carcinogenesis as well [21]. Many currently available research findings point to this kind of interaction in tumors, allowing the identification of inhibitory axes made up of a lncRNA, a sponged miR, and a target mRNA. Since lncRNA and mRNA are both able to bind one or more miR, they are called competing endogenous RNAs (ceRNA) and the intertwined axes sharing one or two members of each axis create a network of cross-interactions collectively called a ceRNA network, or ceRNET [22]. In this perspective, EC is not an exception. Numerous scientific papers have been published, which elaborate on ceRNA interactions in this cancer, and we already summarized the current knowledge in the past [23,24,25,26,27,28,29]; however, a comprehensive and detailed analysis of this topic in recent years and the analysis of EC ceRNET in its entirety is still lacking.

For the purpose of this systematic review, we collected the most up-to-date knowledge on EC-related ceRNETs, revised previous research findings, and used such data to build a hand-curated ceRNET for EC. Our work aims to shed a light on the complexities of ceRNA interactions in this tumor and to aid making accurate predictions for both long-distance interactions and possible future discoveries, eventually making use in the future of adequate bioinformatic tools to speed up discoveries and compare ceRNET from different tumors. Also, we trust that this kind of data will be useful in helping physicians to select appropriate therapies, on a single-patient base, to improve the efficacy of personalized medicine.

## 2. Results

### 2.1. Data Collection

By applying the inclusion and exclusion criteria described in Section 4 (Materials and Methods), we were able to collect data from 172 different bibliographic sources, for a total of 201 ceRNET axes, including partial axes. These results are summarized in Table 1.

Due to their nature and biosynthesis [202] and their official nomenclature (summarized in [203]) and in absence of more specific data, we classified those with a general name and those with the specific 3p or 5p signature as the same miR; for example, we considered miR-101 and miR-101-3p to be the same molecule, as they both derive from the same precursor pre-miR and there is no evidence of sequence differences in the analyzed literature. Instead, miR with both 5p and 3p forms expressed in EC and, thus, potentially those with different targets (they have complementary sequences), were considered as different molecules, for example, miR-129-2-3p and miR-129-5p. Finally, miR, identified by additional letters in their ID, were considered as different miR, as they have slightly different sequences and the research data herein analyzed do not show that they target the same ceRNA; for example, miR-106a and miR-106b were considered different molecules. Consequently, according to these criteria, we considered miR-181a and miR-181a-5p the same molecule but different from miR-181c, so in total, there are three miR forms but only two molecules involved; similar can be said for miR-200 (four forms, three molecules) and miR-27 (four forms, two molecules).

In conclusion, and according to the above-mentioned criteria, this screening allowed us to identify 79 lncRNA, 127 miR, and 140 mRNA targets in EC ceRNET (Table 1).

### 2.2. Data Assembly

We analyzed the axes reported in Table 1 (one ceRNET axis per table row) in search of common elements and joined together the axes using the shared element(s). This allowed us to group these axes in a series of sub-networks of different complexity, that we arbitrarily classified as low (5 subnetworks), medium (7 subnetworks), and high (1 subnetwork) complexity ceRNET. We defined low complexity subnetworks as all those networks with 4 to 5 elements (cells in Table 1) and connecting only 2–3 ceRNET axes (rows in Table 1). All axes with only 2–3 elements (i.e., depicting only 1 axis or 2 axes with a single shared element) are not illustrated. Then, we defined medium complexity subnetworks as all those networks with a number of elements between 6 and 50 and connecting more than 3 axes. Hence, the high complexity subnetwork we identified is defined as the one exceeding medium complexity ceRNET thresholds.

**Figure 1:**
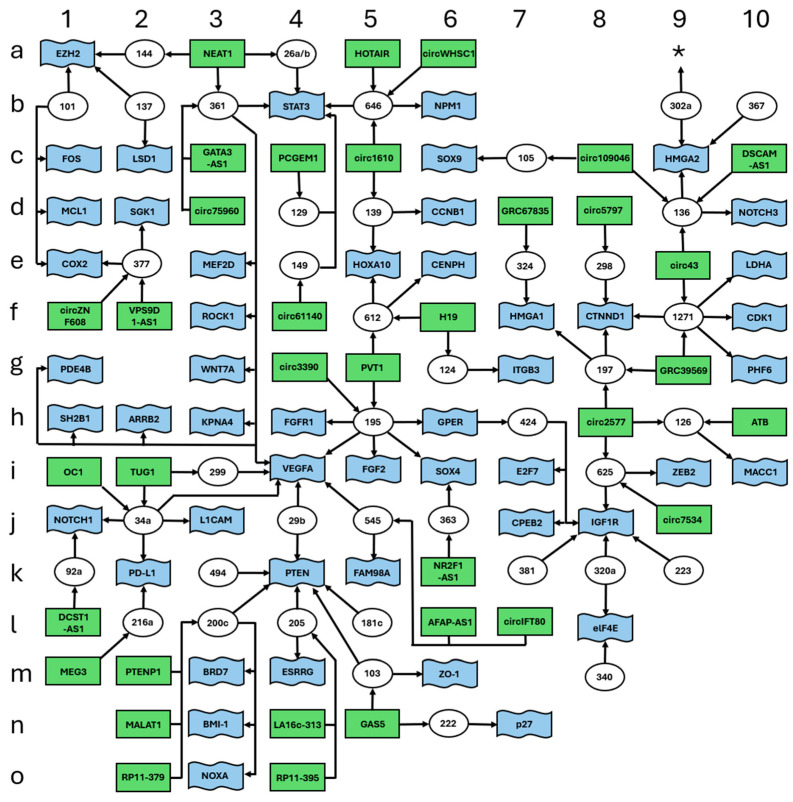
High complexity ceRNET for EC. Data retrieved from Table 1. The figure shows the interconnections of 125 elements, namely 41 miR (white circles), 35 lncRNA (green rectangles), and 49 mRNA targets (blue flags). Arrows indicate the direction of the inhibition, with lncRNA inhibiting miR action via sponging and miR inhibiting target mRNA translation via (mostly) 3′ UTR binding. Letters and numbers surrounding the scheme help find single elements inside the scheme. The asterisk (coordinates: a9) indicates a possible connection with another peripheral branch; see Section 3 for further explanations.

The single high complexity ceRNET we identified (Figure 1) contains a total of 125 elements, and it includes 41 miR (white circles), 35 lncRNA (green rectangles), and 49 mRNA targets (blue flags). These elements were drawn according to a grid so that every element can be easily found by a couple of coordinates, left to right (1–10) and top to bottom (a–o), to allow their easy localization according to such coordinates; numbers and letters surrounding the scheme represent the coordinates. Arrows indicate the direction of the inhibition, with lncRNA inhibiting miR action via sponging and miR inhibiting target mRNA expression via (mostly) 3′ UTR binding. For graphical reasons, the names of some elements in all figures are shrunk to a minimal unambiguous size; thus, for example, “101” (coordinates: b1) indicates miR-101 and circ1610 (coordinates: c5) indicates circ0001610. Full names of the molecules can be found in Table 1.

**Figure 2:**
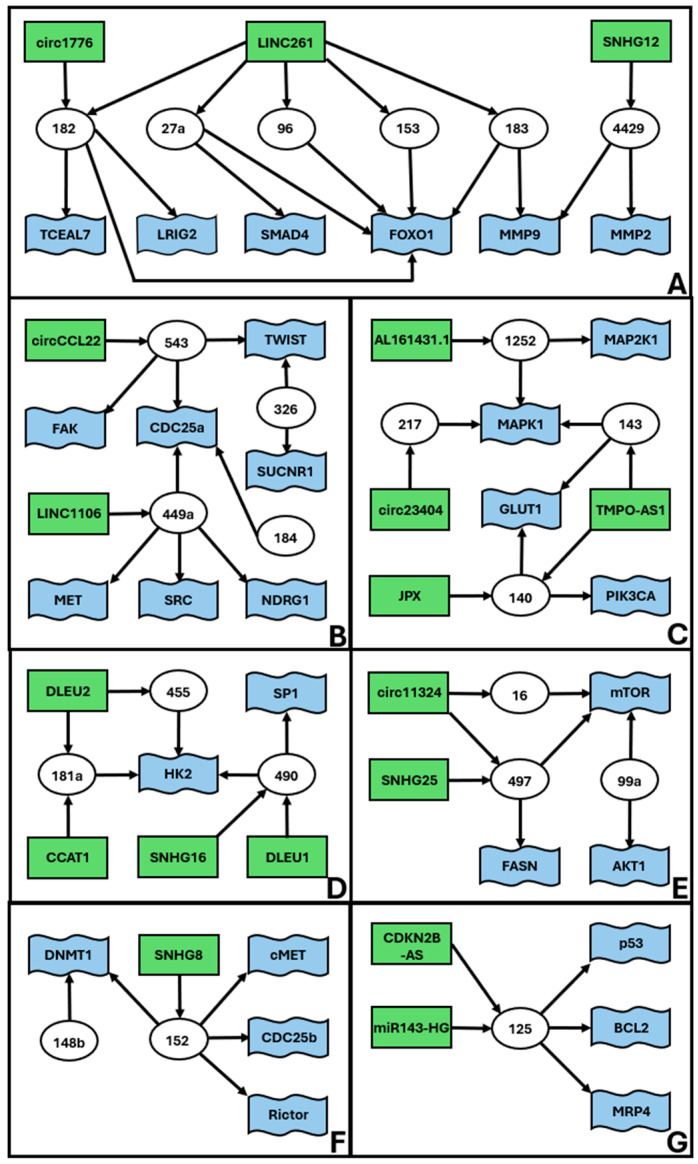
Medium complexity ceRNETs for EC. Data retrieved from Table 1. The figure shows the interconnections of groups of elements, namely miR (white circles), lncRNA (green rectangles), and mRNA targets (blue flags). Arrows indicate the direction of the inhibition, with lncRNA inhibiting miR action via sponging and miR inhibiting target mRNA translation via (mostly) 3′ UTR binding. We define medium complexity ceRNET as all those networks (apparently) unrelated to the main ceRNET (Figure 1) and containing a number of elements between 5 and 50. The identified 7 subnetworks (**A**–**G**) are listed according to their complexity, i.e., number of elements in the ceRNET.

In addition to the high complexity ceRNET, we found a series of smaller medium complexity unconnected subnetworks (Figure 2). These ceRNETs include a number of elements between 15 (Figure 2A) and 6 (Figure 2G).

**Figure 3:**
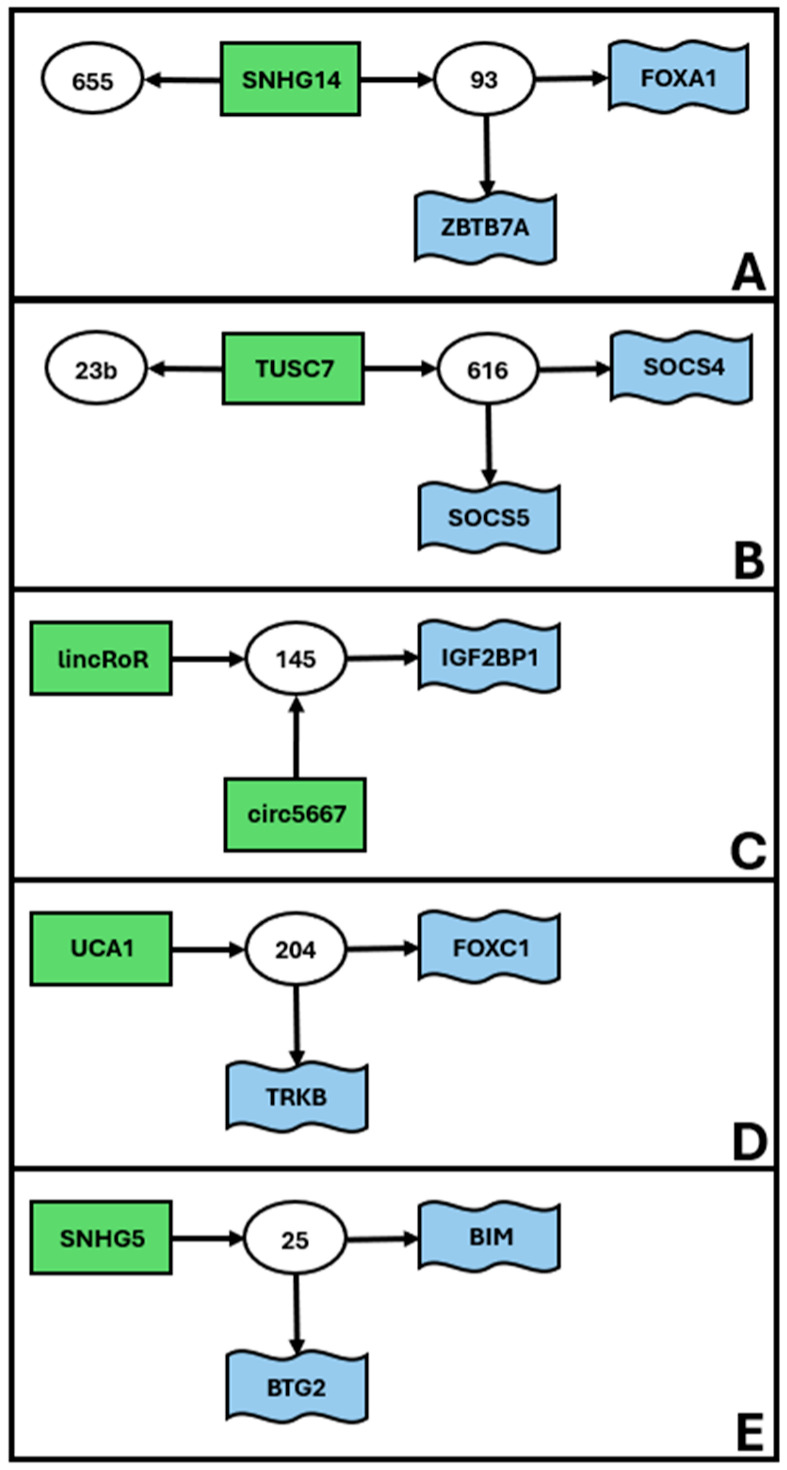
Low complexity subnetworks for EC. Data retrieved from Table 1. The figure shows the interconnections of groups of elements, namely miR (white circles), lncRNA (green rectangles), and mRNA targets (blue flags). Arrows indicate the direction of the inhibition, with lncRNA inhibiting miR action via sponging and miR inhibiting target mRNA translation via (mostly) 3′ UTR binding. We define low complexity ceRNET as all those subnetworks (apparently) unrelated to the main ceRNET (Figure 1) and having 4 to 5 elements, as they connect only 2–3 ceRNET axes. The idenfified 5 subnetworks (**A**–**E**) are listed according to their complexity, i.e., the number of elements in the network. All axes have only 2–3 elements (i.e., depicting only 1–2 axes) and are not reported in figures but can be found in Table 1.

Finally, our search allowed us to identify a series of smaller low complexity unconnected ceRNET (Figure 3), including 4 to 5 elements, apparently not connected with those previously described.

## 3. Discussion

### 3.1. General Considerations

To the best of our knowledge, the present work is the first graphical representation of the functional and experimentally verified networks of EC epigenetic gene expression control. Although far from complete, these schemes provide a good idea as to the complexity of cross-interactions acting at the same time inside an endometrial cell, to maintain its homeostasis. To build these schemes, we manually reviewed the available literature, with stringent selection criteria (see Section 4) to lower the presence of false positives as much as possible. Indeed, especially for medium and high complexity networks, the identified connections provide additional indirect evidence of the robustness of our approach (see also the next paragraph). However, in many cases, these axes had been investigated only in one or two papers, so we encourage other scientists to further validate the connections, especially those which were only inferred, to improve the reliability of our predictions, to expand the general EC ceRNET, and/or to connect the subnetworks we illustrated in Figure 1, Figure 2 and Figure 3.

### 3.2. Using Our ceRNET for Investigating Data Robustness

The very essence of scientific data is well known to reside in its replication. If an experiment fails to be replicated in an independent set-up by other scientists, the most common explanation is that the data are not thoroughly reliable or, in the worst-case scenario, they are either mistaken or misinterpreted. Functional data replication can be obtained in two ways: by replicating the experiment as it is or by finding (hopefully numerous) indirect supporting data. In this report, the use of a luciferase assay has been used as a mandatory selection criterion to verify the physical interaction of any miRNA with a ceRNA (see Section 4). Nonetheless, additional and diverse experimental approaches were used in the research sources we have drawn upon to further validate these findings, such as the use of miR-mimics, or analysis of cells and tissue samples with RT-qPCR and Western blotting. However, other approaches might also be used, such as RNA pull-down or CRISPR screens or the application of -omics data to refine the ceRNET, which would equally substantiate the validity of the luciferase assay. At present, we do not believe that one specific test would suffice to corroborate the luciferase assay result, but a combined use of more approaches would certainly be highly recommended. This would clearly have a positive effect not just on the ceRNET validity but also on the clinical applications derived from its analysis during patients’ diagnosis and prognosis.

If we examine the intricate EC ceRNET depicted in Figure 1, we observe that the majority of its components have been identified in various studies. However, these studies often do not align in their axis descriptions, sharing only individual elements. Within this framework, the presence of miR-195 (coordinates: h5) is highly improbable to be an error or a misinterpretation. The robustness of miR-195 as a central element in EC etiopathogenesis is evident, given its involvement in eight distinct connections sourced from four different literature references (Table 1). Consequently, the study elucidating the interaction between circ_0003390 (coordinates: g4) and miR-195 stands on a solid foundation, even though it is backed by a single bibliographic citation. This long non-coding RNA is in direct association with a sturdy element of the ceRNET, emphasizing its significance. In this context, the relative strength of each component within the complex ceRNET illustrated in Figure 1 can be promptly and effortlessly evaluated based on its interactions with neighboring elements and the extent of research exploring its functions. Thus, any new epigenetic player that will be discovered in the future may be compared to the EC ceRNET reported here to evaluate its affordability on the basis of its possible interactions with known elements of this network. As a mere philosophical exercise, we mined PubMed by using the search string “endometrial cancer AND miR AND retracted” for unverified data on this topic. Our search provided 30 eligible publications (see Section 2—Results, and Section 4—Materials and Methods), describing a total of 27 different miR; of them, 16/27 (ca. 60%) miR are absent in our ceRNETs and 11/30 (ca. 37%) publications did not show a single ceRNET element in common with our reported data (Supplemental Table S1). Of course, these numbers are not compatible with a statistical validation of new ceRNET axes to be discovered in the future, nor is our high complexity network comprehensive enough to make such assumptions; nonetheless, they might provide a hint about those axes that need to be verified with higher-than-average accuracy.

Our work is far from being complete; additional experimental validation is required for those molecules that have only once been identified or only have a limited number of connections. This would also help in expanding the ceRNET through already existing data. In this context, a special case is represented by miR-302a-3p (not included in Figure 1) and miR-302a-5p (coordinates: b9) (Figure 1) (Table 1). As illustrated before, we considered these miRNAs as two different molecules because they have complementary sequences. However, they both come from the same pre-miRNA, and they both are expressed in EC, suggesting a strong functional, spatial, and temporal correlation. In this case, it would be worth exploring the possibility of analyzing if the forked axis NFYA (mRNA)/LINC01016 (lncRNA)/miR-302a-3p/miR-3130-3p could be attached to our high complexity ceRNET at coordinates a9 (asterisk in Figure 1). Finding new EC axes sharing one or more elements between miR-302a-3p and the high complexity ceRNET would solve the problem and would also contribute to further validating our EC networks.

Finally, it is worth pointing out that our methodology is meant to enable the visualization of unexplored axes where all components are already in situ. For instance, to the best of our knowledge, no scholarly reference delineating the lncRNA TUG1 (coordinates: i2) (Figure 1) within the same EC axis as the genes *L1CAM* (j3), *NOTCH1* (j1), or *PD-L1* (k2) exists. Nonetheless, TUG1 is well-known to interact with miR-34a, a microRNA that also regulates the aforementioned trio of genes (Table 1). It is to be expected that analyzing EC samples with impaired TUG1 expression will also result in the deregulation of at least some of these genes. Indeed, connections between TUG1 and either *NOTCH1* or *PD-L1* have already been described in the literature for glioma [204] and hepatocellular carcinoma [205], respectively. Exploring these yet unverified axes will eventually confirm the robustness of our identified ceRNET and inferred connections.

### 3.3. Using Our ceRNET for Investigating Metabolic Changes in EC

In its most basic form, a ceRNET axis allows controlling one target mRNA and thus alters the protein function it encodes by quantitative regulation. In case the target is, for example, an enzyme converting substance A into substance B, an impairment of the enzyme will cause the intracellular depletion of B and a consequent increase in A, a fact known since the experiments made in 1941 by Beadle and Tatum [206]. In those same years, Roger Williams introduced the concept of “metabolic fingerprint” by employing over 200,000 paper chromatograms to demonstrate the high variability of taste thresholds and excretion patterns of various substances among individuals, while remaining constant within individual subjects [207]. Presently, the metabolome is defined as the assemblage of small (<1500 Da) molecule metabolites that delineate a cell, an organ, or an organism, offering a snapshot of the physiological state of a living being [208]. The Human Metabolome DataBase v. 5.0 (https://www.hmdb.ca/, accessed on 25 March 2025) presently encompasses 220,945 metabolite entries comprising both water- and lipid-soluble metabolites, in addition to 8610 protein sequences (comprising enzymes and transporters) associated with these entries. The metabolome is invaluable in discerning diverse conditions impacting the same organ; for instance, in this scenario, to emphasize the distinctions among a healthy endometrium, endometriosis [209], and EC [210,211], as well as aiding in the differential diagnosis of EC [211]. In particular, a systematic Review identified 55 metabolites that hold diagnostic, prognostic, or histotype identification, metastasis, or recurrence risk significance [210]. This underscores the critical role of lipidic metabolites, such as phospholipids, essential for membrane growth during cell division, and those associated with glycolysis. In cancer, glycolysis often supplants oxidative phosphorylation even in aerobic conditions, a phenomenon known as the “Warburg effect” [212]. Another study revealed 111 significantly elevated and 148 reduced metabolites in EC [213]. Numerous findings have pinpointed phosphocholines, acylcholines, carnitines, and other lipid by-products as promising diagnostic biomarkers, alongside various amino acids (as reviewed in [211]).

EC ceRNET facilitates the identification of proteins involved in cancer-altered metabolic pathways and enables the detection of inferred connections between ceRNAs. This capability allows for a more profound analysis of the genetic, molecular, and metabolic status of the patient. Similarly, it offers insights into potential chemotherapy responses, thereby influencing the therapeutic approach. To validate this, we utilized the miRNet 2.0 tool (available at URL: https://www.mirnet.ca/ and accessed on 25 March 2025) [214] with default settings to conduct an enrichment analysis of the ceRNET for EC elements (miRNA, lncRNA, and mRNA targets) listed in Table 1. We applied the minimum network option and conducted gene ontology (GO) analysis, covering the biological process (BP), as well as KEGG pathway enrichment analysis, using the KEGG and GO BP databases within miRNet. The GO and KEGG categories, considered statistical significance at *p* < 0.05, are listed in Supplemental Table S2 in decreasing order of the adj.Pval value, that is, the pValue obtained by standard enrichment analysis based on the hypergeometric tests after adjustment for false discovery rate. As anticipated, the KEGG analysis unveiled eight different malignancies, encompassing chronic myeloid leukemia, prostate cancer, renal cell carcinoma, acute myeloid leukemia, glioma, small cell lung cancer, pancreatic cancer, and non-small cell lung cancer, in the top 20 rankings, in addition to pathways intricately linked to carcinogenesis such as ErbB [215], Wnt [216], and insulin [217], all of which are well-recognized contributors to EC. The inclusion of WNT7a within our principal ceRNET is notable (coordinates: g3). The GO analysis aligns seamlessly with our expectations; as previously mentioned, metabolites exhibit a close association with enzymes and transporters. Among the top 20 outcomes of the GO analysis, 11 pathways are dedicated to the movement of molecules, and notably, “response to ionizing radiation” is also featured, underscoring its relevance to the utilization of radiotherapy in patients. Collectively, these findings underscore the value of EC ceRNET analysis in assessing the metabolic profile of patients, thereby impacting diagnostic, prognostic, and therapeutic decisions.

### 3.4. Using External Data to Expand EC ceRNET

As described in the Results, our work allowed us to identify several minor subnetworks of lower complexity, compared to the one reported in Figure 1. At the moment, these ceRNETs (Figure 2 and Figure 3) do not share elements with each other and should be considered as independent players in EC pathogenesis. However, it is widely recognized that many of the molecules found in EC ceRNET are also deregulated in various other types of cancers. Consequently, by exploring ceRNET axes existing in different cancers yet sharing only partial components with the EC ceRNET, it becomes feasible to integrate smaller subnetworks into larger ones. For instance, miR-143 is a constituent of the moderately intricate EC ceRNET depicted in Figure 2C. In 2017, Wang and collaborators showed that, in bladder cancer (BC), miR-143 is a functional repressor of *IGF-1R* [218] that, in our EC ceRNET, is present in Figure 1 (coordinates: j8) and exhibits the above-mentioned characteristics of robustness. Li and coworkers described the same miR in the same tumor (BC) in 2020 [219]; they report its physical interaction with *COX2* mRNA, another element of our high complexity EC ceRNET (coordinates: e1) (Figure 1). In addition, miR-143-3p interacts, in BC, also with *EZH2* mRNA [220], which in our EC ceRNET is at coordinates a1 (Figure 1) and under the same miR-101 (coordinates: b1) control as *COX2*. Finally, again in BC, miR-143 also interacts with lncRNA UCA1 [221], reported in one of our low-complexity EC subnetworks (Figure 3D). Given that these data are from the same tumor (BC), it is likely that a deeper analysis of BC ceRNET will bring together all these elements, building a ceRNA network of at least medium complexity. This means that, using suitable approaches, i.e., by selecting appropriate elements connected to miR-143, the high complexity EC ceRNET of Figure 1 might be expanded to include the medium complexity one of Figure 2C and, maybe, also the low complexity one reported in Figure 3D.

By harnessing similar tactics, this approach will also enable the direct expansion of our EC ceRNET or increase data robustness by studying its peripheral elements, that is, those with only 1–2 connections, for which their physical interactors are known in other cancers.

### 3.5. Automation of ceRNET Construction Through Artificial Intelligence (AI) and Construction of a Multi-Cancer ceRNET

We are fairly confident that our selection criteria are rigorous enough to construct an economical ceRNET for any known cancer, provided that an ample amount of information (i.e., validated references) is accessible in bibliographic repositories. However, the growing understanding of ceRNET and the emergence of cutting-edge technologies to discover new epigenetic participants will soon render manual ceRNET construction an inconceivable undertaking. In this scenario, AI-driven methodologies could be employed for both the expansion and comparison of ceRNETs. To expand, appropriate cues, aligned with our selection criteria, should empower the AI to recommend to researchers which unexplored axes should be investigated, thereby optimizing the likelihood of yielding a favorable outcome. Furthermore, on a purely theoretical premise at present, we anticipate the future establishment of a “universal tumor ceRNET” where each individual tumor ceRNET could serve as a subnetwork. This would enable the research community to shed a light on the differences among tumors and possibly also the molecular differences of the same tumoral histotype. This will be central to gaining a deeper understanding of the genetic origin of the tumor and allow physicians to select appropriate therapies, on a single-patient basis, to maximize efficacy and minimize side effects, by targeting overlapping or non-overlapping parts of the ceRNET, according to the molecular characterization of the patient’s disease. We also expect that our data, adequately validated and expanded, will also help in deciding the most probable patient’s response to a specific treatment, thus inferring a more accurate prognosis. In this case, AI-based tools will be highly effective in highlighting similarities and differences among such subnetworks and assist physicians in decision-making.

**Figure 4:**
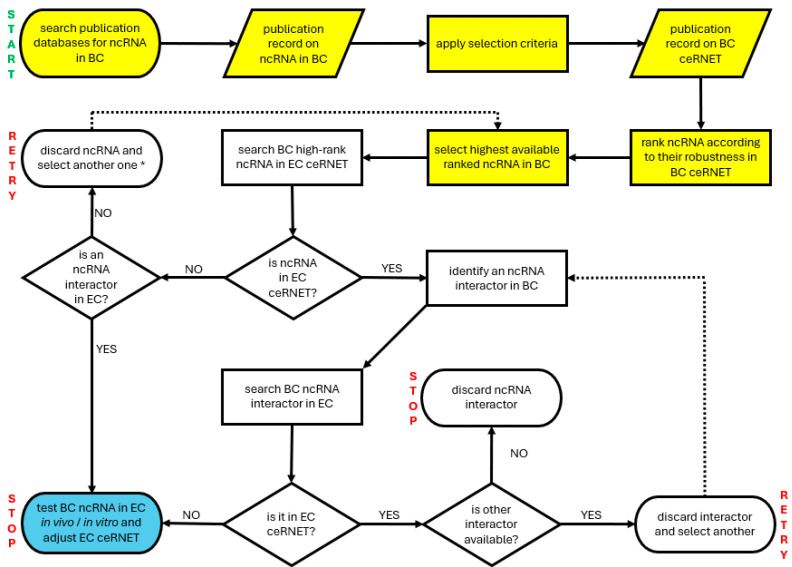
A simplified flowchart illustrating a possible way to instruct an AI tool to expand an existing ceRNET. In this example, the intent is to expand the EC ceRNET using data coming from BC (bladder cancer) ceRNET. START and STOP indicate the start and end points of the algorithm; RETRY indicates where to start over searching. Yellow indicates the preparatory steps required to collect data to be compared (new ceRNET building) if not yet available. Blue indicates the step allowing us to expand/amend/adjust the EC ceRNET. Dotted arrows show where to restart in case of negative results, if possible. See the text for further explanations. Note that this algorithm is inefficient in the case of completely new ceRNET axes (the weak point is indicated by an asterisk in the retry box, second row) because it is based on the comparison of shared ncRNA; in those cases, a different strategy is required, such as starting over using a third tumor.

Considering what we described above (Section 3.4) about BC and miR-143 interactors, it is quite straightforward to foresee possible algorithms for AI-tools to infer the existence of new EC ceRNET axes or at least to expand partial/peripheral portions of the network (Figure 4), eventually leading to additional in vitro and in vivo tests. For example, AI tools could be trained to identify the available literature using the selection criteria described in Section 4, but for a different tumor (for example, BC, but it would work with any cancer). Then, the researcher might prompt the AI to rank those ncRNAs with the higher robustness in BC (on the basis of the number of interactors and on the number of publications in which they appear) and weigh such a list against the one with EC ceRNET elements illustrated in Section 3.2 and reported in Figure 1. This would likely result in two groups of data: (i) a group of BC ncRNAs that are shared and integrated, using the same criteria, with our ceRNET EC (Figure 1) and (ii) BC-specific ncRNAs. In the first case, AI might serve as a means of comparison of shared ncRNA interactors in the two sets (EC and BC) to identify the differences and to verify the robustness of the non-shared elements in BC. Finding a robust interactor in BC that is not present in EC but that targets molecules common to both cancers should represent a viable clue to be tested at the bench (in vitro) or to be sought in EC patients (in vivo), by investigating the possible deregulation of such an element. In the latter case, the investigation of a given robust ncRNA in BC may be used in EC cases, in terms of the presence of its interactors, and then, following a similar procedure, to verify its presence in EC and adjust the EC ceRNET accordingly.

Such a methodological approach can serve as a mere illustration of guiding and training AI tools and does possess its constraints, the primary one being its reliance on the juxtaposition of various ceRNETs, thereby rendering it inadequate for delving into axes that are entirely non-existent within a specific ceRNET. In such instances, alternative approaches can be considered, e.g., acquiring data from a distinct tumor for comparative analysis, the underlying rationale being the improbability of a particular ceRNET axis, along with all its constituents, experiencing exclusive dysregulation in just one type of tumor.

### 3.6. Importance of EC ceRNET for Personalized Therapy: Implications for Precision Medicine

Current knowledge on any cancer-related ceRNET, including EC, is extremely fragmented because most published manuscripts either describe single ceRNET axes or make predictions on many ceRNAs based on bioinformatics only. Thus, it is extremely difficult for physicians to have a general yet thorough overview of the complexity of the interactions inside a tumor hence the difficulty in appropriately assessing a prognosis, choosing a therapy, and predicting, as much as possible, side effects. Our ceRNET representation, as well as any future expansion of it, will hopefully shed light on such aspects and foster informed decision-making. For example, *PTEN* (coordinates: k4) is known to be a frequent central player during sporadic EC formation and development; PTEN protein is involved in the PI3K–PTEN–AKT–mTOR pathway, which regulates cell growth, cell survival, protein synthesis, and metabolism [222]. In this scenario, having a patient that is positive for the alteration of PTEN function should prompt the doctors to also analyze its next interactors—PTEN expression is controlled in EC by at least six different miR (Figure 1), some of which also control the expression of other genes important for EC pathogenesis, such as *VEGFA* [223]. Finding an alteration in *VEGFA* (coordinates: i4) expression should prompt the analysis or its six miR controllers, which in turn regulate the expression of other central EC-related genes, such as *NOTCH1* (j1), *WNT7A* (g3), *STAT3* (b4), *SOX4* (i6), *FGFR1* (h4), or *FGF2* (i5). This targeted molecular investigation is expected to significantly enhance not only the molecular profiling of the patient but also to have a more accurate prognosis and to knowingly decide on a more suitable therapy, not merely based on standard general protocols. For example, knowing that a patient has *PTEN* (Figure 1) or *MAPK1* (Figure 2C) function impaired and knowing that these two genes are also implicated in chemotherapy resistance might drive the physician toward an alternative therapeutic approach.

Our data are derived in most cases from well-characterized patients with overt disease, and available information is not sufficient to assess whether it is theoretically possible to use EC ceRNET for early cancer diagnosis. We already discussed the possibility of using circulating miRNA as EC biomarkers [29]; there, we listed the 16 possible miRNAs potentially useful for this purpose. Quite interestingly, those miRNAs are underrepresented in this report. Only two of them (miR-205 at coordinates l4 and miR-223 at coordinates k9) appear in Figure 1, i.e., the high complexity major ceRNET. In addition, only miR-205 shows the robustness described above, with miR-223 being in a peripheral position (although connected with *IGF1R*, a robust element of the network). Overall, miR-205 seems a good candidate biomarker to detect EC using body fluids, while miR-223 needs further investigation. Of the remaining 14 miRNAs, 8 are completely absent from the present report. One possibility is that these miRNAs will join our ceRNET if specifically tested in suitable samples and with appropriate techniques (i.e., luciferase assay). Another possible explanation is that those eight miRNAs are not part of the EC ceRNET but might either fulfill other tasks or be a byproduct of general cellular metabolism deregulation, a typical cancer feature. The possible role of the remaining six miRNAs, namely miR-27a (Figure 2A), miR-449 (Figure 2B), miR-99a (Figure 2C), miR-204 (Figure 3D), miR-141, and miR-200a (no figure), is more puzzling. We suggest two possible explanations. First, the medium/low complexity EC ceRNETs containing them will eventually be joined to the main one, and in this case, their role in the EC major ceRNET will be clear. But another intriguing possibility arises if these minor ceRNET remain independent. In this case, it should be possible to hypothesize that more than one molecular mechanism could be involved in EC etiopathogenesis or that these minor networks describe temporally separated (early/transient markers?) events from the main pathway depicted in the remaining major ceRNET. Of course, in this phase of the research, this should be considered only a speculative plot, but from this perspective, it becomes crucial to investigate those minor networks to verify their independence from the main one.

We checked the website ClinicalTrials.gov (last accession: 15 January 2025), looking for any ncRNA listed in Table 1 and currently tested in therapy, either as a target or as a drug; we discarded those trials investigating them as mere biomarkers. None of the lncRNAs in Table 1 are present in the database. As for the miR, only two are present, namely miR-155 (NCT02580552, NCT03837457, NCT03713320) and miR-34a (NCT01829971, NCT02862145). To date, miR-155 seems to be a marginal player in EC (Table 1) while miR-34a is part of our major ceRNET (coordinates: j2) (Figure 1), where it shows interactions with two lncRNAs and four mRNAs. Functionally, this miR is also quite near to *PTEN* (k4) and directly connected to both *VEGFA* (i4) and *NOTCH1* (j1) discussed above, making it an amenable EC therapy target. Unfortunately, both clinical trials involving a miR-34 mimic (MRX34) were aimed at testing its efficacy as a drug in various tumors including viral-related hepatocellular carcinoma, melanoma (non-cutaneous, excluding uveal), small cell lung cancer, triple-negative breast cancer, sarcoma, and bladder, renal, and ovarian cancers [224,225,226] but not EC. Beyond miR-34, our schemes point to additional molecules that could be tested in this perspective but for which no clinical trial is currently running or even planned.

### 3.7. Legal and Ethical Aspects of Using ceRNET in Cancer Therapy

As research illuminates EC ceRNET, researchers and doctors will better outline innovative therapeutic strategies to strengthen, optimize, or replace current approaches. ceRNETs help clarify long-term interactions for personalized medicine [227,228]. Personalized medicine is based on individual patient characteristics for effective treatment. Novel care approaches promise improved effectiveness and reduced toxicity. Legal and ethical implications of personalized medicine must be addressed [229,230,231]. Governance of personalized medicine protocols should prioritize privacy, fairness, and non-discrimination. Techniques of personalized medicine will expand with ethical guidelines. Multidisciplinary efforts are needed for the development of personalized medicine, which will evolve alongside big data analysis and artificial intelligence. Management of personalized medicine requires a diverse skill set beyond traditional medical professionals [27,232]. Such novel avenues will develop alongside other high-potential technologies such as big data analysis and artificial intelligence [27]. Such a highly complex and multi-faceted form of management and implementation cannot therefore rely solely on medical professionals or lab technicians in their traditional form and conventional skill-sets and training [232]. The tremendous still partly untapped potential of personalized approaches to cancer care had already been highlighted in 2014 by the European Society for Medical Oncology (ESMO) [229] in a guide characterizing personalized medicine as the future of cancer medicine. Yet, with great power and potential comes great responsibility: implications from novel approaches must be addressed to establish well-defined criteria based on fairness and equal opportunities while preventing discrimination [230]. While a global review of personalized medicine projects and initiatives is too extensive for this article, it is worth mentioning ongoing EU undertakings [233,234,235] taking into account future cancer diagnostics or therapeutics, translational research, the transition from an “organ-centric” notion of therapeutic and diagnostic pathways toward thorough molecular analysis [236], i.e., the ultimate driver of personalized approaches, in spite of the still relatively high degree of uncertainty in terms of outcomes [237]. European institutions recognize the breakthrough in future cancer diagnostics, translational research, and personalized approaches [238]. The key to personalized medicine lies in the synergy between health data and new technologies, leading to effective treatment strategies. This approach can have wide-ranging positive impacts on public health and healthcare systems. In 2022, the EU introduced measures to leverage innovative technologies like AI, big data, and genomics. Ongoing projects like PCM4EU and EUCAIM [239,240,241] aim to advance personalized medicine using novel tools. The EUCAIM platform is set to be fully released by December 2025. The EU aims to establish a federated European cancer images data repository by 2026. The Partnership on Personalized Medicine, launched in 2023 [242], focuses on involving public and private stakeholders and relying partly on funding allocated by Horizon Europe, i.e., the EU’s chief funding program aimed at furthering research and development. Harmonizing personalized medicine strategies and policies is, in fact, crucial for equitable access and to prevent “healthcare travels”.

#### Medicolegal Tenability Needs Updated Frames of Reference

The framing and acknowledgement of international standards are crucial in medicolegal aspects. Personalized medicine’s evolving nature may lead to uncertainty and malpractice risks [243]. Genetic malpractice is a relatively recent notion that entails the doctors’ failure or negligence to implement genetic testing or failing to adequately draw upon and interpret its findings. There is, in fact, no sufficient degree of uniformity in terms of assessing the values, scope, and timing of genetic testing or even on whether such testing ought to be executed at all, and under what conditions [243,244,245,246]. Data on such novel litigation profiles are still inconclusive, and it is still unclear how they may impact healthcare delivery and management [247]. Compliance with legislative standards and evidence-based guidelines is essential for genetic testing guidelines and needs to be thoroughly documented throughout the procedures, as the onus to prove adherence to guidelines and best practices rests on doctors and healthcare professionals, particularly under civil law statutes [248,249]. A high level of objectivity, rooted in validated scientific and clinical elements, will therefore be key to shaping legal and judicial trends and court rulings, thus shielding physicians and facilities from malpractice lawsuits, which are often frivolous and can unduly burden healthcare systems, much to the detriment of public health and sustainability.

## 4. Materials and Methods

We searched the PubMed database (URL: https://pubmed.ncbi.nlm.nih.gov/ accessed on 1 October 2024) using, as keywords, the terms “endometrial cancer” in combination with the following terms (Boolean operator: AND): (a) non-coding RNA; (b) ncRNA; (c) miRNA; (d) miR; (e) lncRNA; (f) lincRNA; (g) circRNA; (h) ceRNA; and (i) ceRNET. The literature collection was performed between 1 and 31 October 2024, with no time limit. Every article was analyzed manually by at least two researchers; no AI-based tool was used during the process, for either data selection or analysis.

The exclusion criteria were the following: (a) articles dealing with bioinformatics predictions but not showing bench data on the physical interaction between any given miR and at least one ceRNA; (b) studies involving only animal models (mostly, *M. musculus*); (c) indirect interactions or negative correlations not supported by data showing physical interactions between a miR and at least one ceRNA; (d) partial axes involving only a correlation between the expression of an lncRNA and a mRNA. Specifically, an exclusion criterium (d) was adopted because it has been repeatedly shown in the literature that lncRNA may alter target gene expression not only through a ceRNET but also through many additional and independent mechanisms, including chromatin remodeling, transcriptional control, and post-transcriptional processing [250], the formation of molecular scaffolds, bringing together different proteins to assemble functional complexes [251], the influence on mRNA stability and translation by binding to complementary sequences or interaction with RNA-binding proteins [251,252,253], and the formation of nuclear bodies and the maintenance of nuclear architecture [254]. In addition, the manuscripts we excluded were excluded by applying criterion (d) in most cases, which did not report an explanation about the described correlation.

The inclusion criteria were the following: (a) use of human specimens, either bioptic materials and/or EC cell lines; (b) use of the luciferase assay (mandatory criterium) and, possibly, other molecular biology approaches (including, but not limited to, miR-mimics, RT-qPCR, Western blotting; we considered these as optional criteria, meaning that the absence of these experimental techniques did not impede us to include the manuscript in our selected list in Table 1) supporting the functional correlation and the physical interaction between any given miR and at least one ceRNA; (c) complete axes description, involving the physical interaction between miR and lncRNA on the one side, and miR and mRNA on the other side; and (d) partial axes involving at least one miR and at least one ceRNA, provided that they satisfy both criteria (a) and (b).

**Figure 5:**
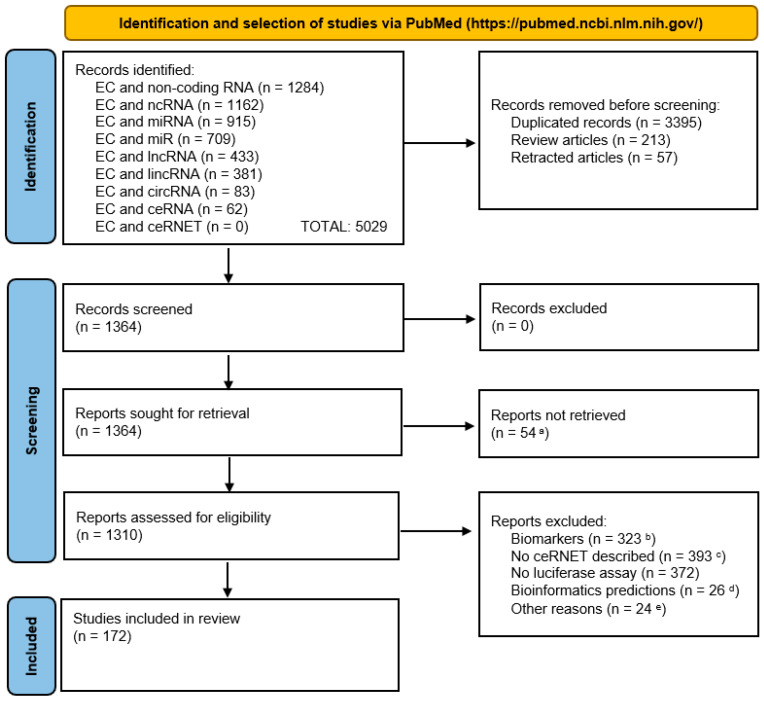
PRISMA flow diagram showing the bibliographic selection criteria applied in this report. For graphical reasons, we abbreviated “endometrial cancer” as “EC”. Notes—a: manuscript not available or manuscript in non-English language; b: manuscripts dealing with the use of ncRNA only as cancer biomarkers; c: no ceRNET axis described (manuscript only focusing on lncRNA) or description of only lncRNA plus mRNA (no miRNA); d: works based only or mostly on bioinformatics predictions with no physical interaction investigated; e: numerically minor reasons, such as the use of non-human samples, unclear methodology, not peer-reviewed papers (e.g., those in the bioRxiv repository). We built the diagram using the PRISMA 2020 flow diagram template distributed in accordance with the terms of the Creative Commons Attribution (CC BY 4.0) license, which permits others to distribute, remix, adapt, and build upon this work, for commercial use, provided the original work is properly cited; the website used as a source of the template is accessible at URL https://www.prisma-statement.org/prisma-2020-flow-diagram (accessed on: 18 March 2025).

A summary of the selection process is illustrated in the PRISMA flow diagram below (Figure 5).

This systematic review has been registered in PROSPERO (ID: PROSPERO 2025 CRD420251035222).

## 5. Conclusions

We used the available literature (Figure 5) to build a hand-curated ceRNET for EC. We identified a total of 217 elements (71 miR, 59 lncRNA and 87 mRNA) grouped in 13 ceRNET, of which 1 is high complexity (Figure 1), 7 is medium complexity (Figure 2), and 5 is low complexity (Figure 3), plus 8 miR, 20 lncRNA, and 53 mRNA, which belong to single axes and are not included in the above-mentioned ceRNET (Table 1). All interactions reported in our schemes represent physical interactions among coding and non-coding RNAs and have been validated by luciferase assay (mandatory) plus additional molecular experiments (optional). These data allowed us to identify 13 subnetworks that, potentially, could be joined into a major EC ceRNET. On the same basis, we foresee that similar work could be conducted for other types of cancer—possibly all, depending on data availability—and that all these ceRNET might, in future, be part of one very large and complex “general tumor ceRNET” that might be used as a reference for finding new cancer players for a more accurate prognosis and therapeutic strategies for cancer patients. We conclude this report by emphasizing that our data represent the starting, not the ending, point of EC ceRNET identification and characterization; although we used stringent selection criteria to build them, our networks need additional experimental testing for both their validation and expansion.

## Figures and Tables

**Table 1:** List of regulatory axes in EC. Each axis (a row in the table) is defined as a group of three elements, a lncRNA (column 2), and a mRNA (column 3) competing for the binding of the same microRNA (column 1). Data are ordered according to column 1. Undefined means that in the relevant publication (column 4), only two members of the axis have been described (partial axes). Column 5 indicates where the axis is illustrated in figures; it is reported in figures only if its ceRNET reaches at least four elements and two interconnected axes; if not, we report “no” in column 5. Asterisks indicate a putative additional connection in Figure 1 (see Section 3.2).

miRNA	lncRNA	mRNA Target	Reference	Figure
miR-101	undefined	COX2	[30]	1
miR-101	undefined	EZH2	[31]	1
miR-101	undefined	FOS	[31]	1
miR-101	undefined	MCL-1	[31]	1
miR-101-3p	undefined	EZH2	[32]	1
miR-103	GAS5	PTEN	[33]	1
miR-103	undefined	ZO-1	[34]	1
miR-105	circ_0109046	SOX9	[35]	1
miR-106a	undefined	BCL2L11	[36]	no
miR-106b	undefined	p21	[37]	no
miR-107-5p	undefined	ERα	[38]	no
miR-10b	undefined	HOXB3	[39]	no
miR-1227	circ_TNFRSF21	MAPK13	[40]	no
miR-124-3p	H19	ITGB3	[41]	1
miR-1252-5p	AL161431.1	MAP2K1	[42]	2C
miR-1252-5p	AL161431.1	MAPK1	[42]	2C
miR-125a	miR143HG	p53	[43]	2G
miR-125a-5p	CDKN2B-AS	BCL2	[44]	2G
miR-125a-5p	CDKN2B-AS	MRP4	[44]	2G
miR-126	ATB	undefined	[45]	1
miR-126-5p	circ_0002577	MACC1	[46]	1
miR-1271	undefined	CDK1	[47]	1
miR-1271	undefined	LDHA	[48]	1
miR-1271-5p	circ_0000043	CTNND1	[49]	1
miR-1271-5p	circ_0039569	PHF6	[50]	1
miR-129-2-3p	XIST	CCP110	[51]	no
miR-129-5p	PCGEM1	STAT3	[52]	1
miR-130b	undefined	ZEB1	[53]	no
miR-132-3p	undefined	MTFR2	[54]	no
miR-134	undefined	POGLUT1	[55]	no
miR-136	circ_0000043	NOTCH3	[56]	1
miR-136	circ_0109046	HMGA2	[57]	1
miR-136-5p	DSCAM-AS1	undefined	[58]	1
miR-137	undefined	EZH2	[59]	1
miR-137	undefined	LSD1	[59]	1
miR-139-5p	circ_0001610	CCNB1	[60]	1
miR-139-5p	undefined	HOXA10	[61]	1
miR-140	TMPO-AS1	GLUT1	[62]	2C
miR-140-3p	JPX	PIK3CA	[63]	2C
miR-141-3p	MIR22HG	DAPK1	[64]	no
miR-143	TMPO-AS1	GLUT1	[62]	2C
miR-143	undefined	MAPK1	[65]	2C
miR-144-3p	NEAT1	EZH2	[66]	1
miR-145	linc-RoR	undefined	[67]	3C
miR-145-5p	circ_0005667	IGF2BP1	[68]	3C
miR-148b	undefined	DNMT1	[69]	2F
miR-149-5p	circ_0061140	STAT3	[70]	1
miR-152	SNHG8	c-MET	[71]	2F
miR-152	undefined	CDC25B	[72]	2F
miR-152	undefined	DNMT1	[73]	2F
miR-152	undefined	Rictor	[73]	2F
miR-153	LINC00261	FOXO1	[74]	2A
miR-155	undefined	AGTR1	[75]	no
miR-15a-5p	undefined	WNT3A	[76]	no
miR-16-5p	circ_0011324	mTOR	[77]	2E
miR-181a	DLEU2	HK2	[78]	2D
miR-181a-5p	CCAT1	undefined	[79]	2D
miR-181c	undefined	PTEN	[80]	1
miR-182	circ_0001776	LRIG2	[81]	2A
miR-182	LINC00261	FOXO1	[74]	2A
miR-182	undefined	TCEAL7	[82]	2A
miR-183	LINC00261	FOXO1	[74]	2A
miR-183	undefined	MMP9	[83]	2A
miR-184	undefined	CDC25A	[84]	2B
miR-191	undefined	TET1	[85]	no
miR-192-5p	undefined	ALX1	[86]	no
miR-195	undefined	GPER	[87]	1
miR-195	undefined	SOX4	[88]	1
miR-195-5p	circ_0003390	VEGFA	[89]	1
miR-195-5p	PVT1	FGF2	[90]	1
miR-195-5p	PVT1	FGFR1	[90]	1
miR-196a-5p	LOC134466	TAC1	[91]	no
miR-197	circ_0002577	CTNND1	[92]	1
miR-197	circ_0039569	HMGA1	[93]	1
miR-200a	undefined	FOXA2	[94]	no
miR-200b	undefined	TIMP2	[95]	no
miR-200c	MALAT1	undefined	[96]	1
miR-200c	PTENP1	PTEN	[97]	1
miR-200c	undefined	BMI-1	[98]	1
miR-200c	undefined	BRD7	[99]	1
miR-200c-3p	RP11-379k17.4	NOXA	[100]	1
miR-204	undefined	FOXC1	[101]	3D
miR-204-5p	UCA1	undefined	[102]	3D
miR-204-5p	undefined	TrkB	[103]	3D
miR-205	undefined	ESRRG	[104]	1
miR-205	undefined	PTEN	[105]	1
miR-205-5p	LA16c-313D11.11	PTEN	[106]	1
miR-205-5p	RP11-395G23.3	PTEN	[107]	1
miR-206	undefined	HDAC6	[108]	no
miR-210-3p	undefined	RUNX1T1	[109]	no
miR-214	DANCR	undefined	[110]	no
miR-21-5p	undefined	SOX17	[111]	no
miR-216a	MEG3	PD-L1	[112]	1
miR-217	circ_0023404	MAPK1	[113]	2C
miR-218	undefined	ADD2	[114]	no
miR-222-3p	GAS5	p27	[115]	1
miR-223	undefined	IGF-1R	[116]	1
miR-23b	TUSC7	undefined	[117]	3B
miR-23c	LINC01410	CHD7	[118]	no
miR-25	undefined	BIM	[37]	3E
miR-25-3p	SNHG5	BTG2	[119]	3E
miR-26a/b-5p	NEAT1	STAT3	[120]	1
miR-27a	LINC00261	FOXO1	[74]	2A
miR-27a-5p	undefined	SMAD4	[121]	2A
miR-27b	RBAT1	undefined	[122]	no
miR-27b-3p	undefined	MARCH7	[123]	no
miR-28-5p	LOXL1-AS1	RAP1B	[124]	no
miR-298	circ_0005797	CTNND1	[125]	1
miR-299	TUG1	VEGFA	[126]	1
miR-29a-3p	circ_FOXO3	HDAC4	[127]	no
miR-29b	undefined	PTEN	[128]	1
miR-29b	undefined	VEGFA	[129]	1
miR-302a-3p	LINC01016	NFYA	[130]	*
miR-302a-5p	undefined	HMGA2	[131]	1
miR-30c	undefined	MTA1	[132]	no
miR-3130-3p	LINC01016	NFYA	[130]	*
miR-320a	undefined	IGF-1R	[133]	1
miR-320a	undefined	eIF4E	[134]	1
miR-324-5p	circ_0067835	HMGA1	[135]	1
miR-326	undefined	SUCNR1/GPR91	[136]	2B
miR-326	undefined	TWIST1	[137]	2B
miR-329-3p	undefined	E2F1	[138]	no
miR-335	undefined	RBM10	[139]	no
miR-340-5p	undefined	eIF4E	[134]	1
miR-34a	OC1	PD-L1	[140]	1
miR-34a	undefined	L1CAM	[141]	1
miR-34a	undefined	NOTCH1	[142]	1
miR-34a-5p	TUG1	VEGFA	[126]	1
miR-34c	undefined	E2F3	[143]	no
miR-34c	undefined	IL-6R	[144]	no
miR-361	GATA3-AS1	ARRB2	[145]	1
miR-361	NEAT1	KPNA4	[146]	1
miR-361	NEAT1	MEF2D	[146]	1
miR-361	NEAT1	PDE4B	[146]	1
miR-361	NEAT1	ROCK1	[146]	1
miR-361	NEAT1	STAT3	[146]	1
miR-361	NEAT1	VEGFA	[146]	1
miR-361	NEAT1	WNT7A	[146]	1
miR-361-3p	circ_0075960	SH2B1	[147]	1
miR-363	NR2F1-AS1	SOX4	[148]	1
miR-367-3p	undefined	HMGA2	[131]	1
miR-372	undefined	RhoC	[149]	no
miR-377-3p	circ_ZNF608	COX2	[150]	1
miR-377-3p	VPS9D1-AS1	SGK1	[151]	1
miR-378a-3p	BLACAT2	YY1	[152]	no
miR-381	undefined	IGF-1R	[153]	1
miR-409	undefined	SMAD2	[154]	no
miR-424	undefined	CPEB2	[155]	1
miR-424	undefined	E2F7	[156]	1
miR-424	undefined	GPER	[157]	1
miR-424	undefined	IGF-1R	[158]	1
miR-4429	SNHG12	MMP2	[159]	2A
miR-4429	SNHG12	MMP9	[159]	2A
miR-449a	LINC01106	MET	[160]	2B
miR-449a	undefined	CDC25A	[161]	2B
miR-449a	undefined	NDRG1	[162]	2B
miR-449a	undefined	SRC	[163]	2B
miR-455	DLEU2	HK2	[78]	2D
miR-485-5p	LINC01224	AKT3	[164]	no
miR-486-5p	undefined	MARK1	[165]	no
miR-490	DLEU1	SP1	[166]	2D
miR-490-3p	SNHG16	HK2	[167]	2D
miR-494-3p	undefined	PTEN	[168]	1
miR-495	undefined	PIK3R1	[169]	no
miR-497-5p	circ_0011324	mTOR	[77]	2E
miR-497-5p	SNHG25	FASN	[170]	2E
miR-498-5p	HOXB-AS3	ADAM9	[171]	no
miR-505	undefined	TGFA	[172]	no
miR-520h	circ_0001860	SMAD7	[173]	no
miR-522	undefined	MAOB	[174]	no
miR-543	circ_CCL22	CDC25A	[175]	2B
miR-543	undefined	FAK	[176]	2B
miR-543	undefined	TWIST1	[177]	2B
miR-545-3p	AFAP-AS1	VEGFA	[177]	1
miR-545-3p	circ_IFT80	FAM98A	[178]	1
miR-589-5p	undefined	TRIP6	[179]	no
miR-6076	CHL1-AS1	CHL1	[180]	no
miR-612	H19	HOXA10	[181]	1
miR-612	PVT1	CENP-H	[182]	1
miR-616	TUSC7	SOCS4	[183]	3B
miR-616	TUSC7	SOCS5	[184]	3B
miR-625	circ_0007534	ZEB2	[184]	1
miR-625-5p	circ_0002577	IGF-1R	[185]	1
miR-626	circ_0000437	CDKN1B	[186]	no
miR-636	MONC	GLCE	[187]	no
miR-638	undefined	MEF2C	[188]	no
miR-646	circ_0001610	STAT3	[189]	1
miR-646	circ_WHSC1	NPM1	[190]	1
miR-646	HOTAIR	NPM1	[191]	1
miR-652	undefined	RORA	[192]	no
miR-655-3p	SNHG14	undefined	[193]	3A
miR-7-2-3p	BMPR1B-AS1	DCLK1	[194]	no
miR-876-3p	circ_NAB1	CDKN3	[195]	no
miR-92a-3p	DCST1-AS1	NOTCH1	[196]	1
miR-93	undefined	FOXA1	[197]	3A
miR-93-5p	SNHG14	ZBTB7A	[198]	3A
miR-940	undefined	MRVI1	[199]	no
miR-944	undefined	CADM2	[200]	no
miR-96	LINC00261	FOXO1	[74]	2A
miR-99a	undefined	AKT1	[201]	2E
miR-99a	undefined	mTOR	[201]	2E

## Data Availability

The original contributions presented in this study are included in the article/Supplementary Materials. Further inquiries can be directed to the corresponding author.

## Supplementary Materials

The following supporting information can be downloaded at https://www.mdpi.com/article/10.3390/ncrna11030034/s1: Table S1: Retracted articles according to PubMed search: “endometrial cancer AND miR AND retracted” performed on 17 January 2025; Table S2: KEGG and GO analysis for the metabolic changes in EC linked to the data reported in Table 1.
