# Supplementary material for: Building a Hand-Curated ceRNET for Endometrial Cancer, Striving for Clinical as Well as Medicolegal Soundness: A Systematic Review
Source: Noncoding RNA. 2025 Apr 30;11(3):34. doi: 10.3390/ncrna11030034 (PMC12101250; doi:10.3390/ncrna11030034)
Supplement: Supplementary file 1 [file ncrna-11-00034-s001.zip › Table S1.pdf]

Table S1. Retracted articles according to PubMed search: “endometrial cancer miR retracted” performed on January 17, 2025. Dashes indicate unavailable data.

doi	miR	lncRNA	mRNA	presence in Table 1
10.3233/CBM-170620	101	---	mTOR	both
10.1080/21691401.2019.1617727	124-3p	OGFRP1	SIRT1	miR only (fig 1)
10.3892/ol.2015.4001	126	---	IRS1	miR only (fig 1)
10.1089/dna.2018.4441	142	---	CCND1	no
10.1016/j.amjms.2022.02.011	144-3p	---	PRR11	miR only (fig 1)
10.1002/cncr.25944	145	---	OCT4	miR only (fig. 3c)
10.3727/096504018X15202988139874	148b	---	ERMP1	miR only (fig 2f)
10.1177/1533033820967462	149-3p	HOXB-AS1	Wnt10b	no
10.1080/15384101.2021.1941611	15b-3p	---	KLF2	no
10.1002/jcb.26763	183	---	CPEB1	miR only (fig 2a)
10.3892/ijmm.2018.3853	183-5p	---	ezrin	miR only (fig 2a)
10.1155/2022/2398101	195-5p	---	JAK2	miR only (fig 1)
10.1042/BSR20190680	202	---	FGF2	gene only (fig 1)
10.3802/jgo.2023.34.e11	202-5p	circ75960	CTNND1	gene and lncRNA but on different rows
10.1080/15384101.2019.1648958	20b-5p	H19	AXL, HIF-1a	H19 only (fig 1)
10.1155/2023/9758785	21-5p	NBAT-1	PTEN	gene and miR; miR not present in figures
10.1186/s13048-020-00639-2	216a	CTBP1-AS2	PTEN	gene and miR but on different rows
10.3233/CBM-170388	216b	CCAT2	BCL-2	gene only (fig 2g)
10.1186/s11671-021-03640-w	216b	Linc01354	KRAS	no
10.1177/0963689721989616	219-5p	Linc00461	COX-2	gene only (fig 1)
10.3892/mmr.2014.2123	22	---	MMP9, MMP22	genes only (fig 2a)
10.1080/15384101.2018.1475829	29a-5p	---	TPX2	no
10.2147/CMAR.S174889	365	---	FOS, EZH2	genes only (fig 1)
10.1002/jcb.28149	373	---	LATS2	no
10.3892/or.2020.7691	376a-3p	TTN-AS1	PUM2	no
10.1080/15384101.2020.1757936	516b	Linc01123	KIF4a	no
10.3389/pore.2021.1609761	543	---	MAPK1	miR (fig. 2b), gene (fig. 2c)
10.1155/2022/7918596	641		AP1G1	no
10.1186/s12957-023-03114-6	7-5p	SOX21-AS1	RAF1	no
10.3892/etm.2019.7713	873		HDGF	no
